# Supplementary material for: Intersection passing strategies for human-driven and autonomous vehicles in mixed traffic using DEA
Source: PLoS One. 2025 Apr 29;20(4):e0321566. doi: 10.1371/journal.pone.0321566 (PMC12040198; doi:10.1371/journal.pone.0321566)
Supplement: Attachment — Submitted filename: Response to Reviewers.docx [file pone.0321566.s002.docx]

**Response to Reviewers**

**Manuscript number:** PONE-D-24-52478

**Title:** Intersection passing strategies for human-driven and autonomous vehicles in mixed traffic using DEA

Dear editor and reviewers,

We are very grateful for your constructive comments and suggestions for our manuscript entitled “Intersection passing strategies for human-driven and autonomous vehicles in mixed traffic using DEA” (ID: PONE-D-24-52478). As you are concerned, there are several problems that need to be addressed. According to your nice suggestions, we have made extensive corrections to our previous manuscript, the detailed corrections are listed below.

We sincerely thank the editor and all reviewers for their valuable feedback that we have used to improve the quality of our manuscript. The reviewer comments are laid out below in italicized font and specific concerns have been numbered. Our response is given in normal font and changes/ additions to the manuscript are given in the yellow text.

Respond to the editor’s comments

***Q1:*** *Please ensure that your manuscript meets PLOS ONE's style requirements, including those for file naming.*

**Response 1:** Thank you for your reminder. I have carefully revised my manuscript according to the PLOS ONE template to ensure it meets the required formatting standards.

***Q2:*** *Please amend your list of authors on the manuscript to ensure that each author is linked to an affiliation. Authors’ affiliations should reflect the institution where the work was done.*

**Response 2:** Thank you for your reminder. I have ensured that each author is linked to their corresponding affiliation.

***Q3:*** *Please note that PLOS ONE has specific guidelines on code sharing for submissions in which author-generated code underpins the findings in the manuscript. In these cases, we expect all author-generated code to be made available without restrictions upon publication of the work.*

**Response 3:** Thank you for your valuable feedback. This study does not involve any code, so no code has been uploaded.

***Q4:*** *Thank you for stating the following financial disclosure:*

*[This research was funded by the Humanities and Social Sciences Research Planning Fund from the Ministry of Education of China, Grant No. 23YJAZH122. It was conducted as part of the project titled "Safety Assessment and Efficiency Optimization of Road Intersections in Mixed Traffic Environments of Manual and Autonomous Driving."].*

*Please state what role the funders took in the study. If the funders had no role, please state: ""The funders had no role in study design, data collection and analysis, decision to publish, or preparation of the manuscript.""*

*If this statement is not correct you must amend it as needed.*

**Response 4:** Apologies for the omission. The funder provides financial support for the research project. I have updated the funder role statement in the cover letter.

***Q5:*** *Thank you for stating the following in the Acknowledgments Section of your manuscript:*

*[This research was funded by the Humanities and Social Sciences Research Planning Fund from the Ministry of Education of China, Grant No. 23YJAZH122. It was conducted as part of the project titled "Safety Assessment and Efficiency Optimization of Road Intersections in Mixed Traffic Environments of Manual and Autonomous Driving." The views and conclusions expressed in this paper are solely those of the authors.]*

*We note that you have provided funding information that is not currently declared in your Funding Statement. However, funding information should not appear in the Acknowledgments section or other areas of your manuscript. We will only publish funding information present in the Funding Statement section of the online submission form.*

*Please remove any funding-related text from the manuscript and let us know how you would like to update your Funding Statement. Currently, your Funding Statement reads as follows:*

*[This research was funded by the Humanities and Social Sciences Research Planning Fund from the Ministry of Education of China, Grant No. 23YJAZH122. It was conducted as part of the project titled "Safety Assessment and Efficiency Optimization of Road Intersections in Mixed Traffic Environments of Manual and Autonomous Driving."]*

**Response 5:** Apologies for this issue. I have removed the funding statement from the manuscript and included it in the cover letter.

***Q6:*** *We note that your Data Availability Statement is currently as follows: [All relevant data are within the manuscript and its Supporting Information files.]*

*Please confirm at this time whether or not your submission contains all raw data required to replicate the results of your study. Authors must share the “minimal data set” for their submission. PLOS defines the minimal data set to consist of the data required to replicate all study findings reported in the article, as well as related metadata and methods*

**Response 6:** Thank you for your valuable suggestion. The data used in this study are assumed values for explaining the research content and verifying the feasibility of the proposed method. These data have been fully presented in Tables 5, 8, and 13 of the manuscript.

Respond to additional editor comments:

***Q1:*** *Justify model assumptions and parameters. The theoretical projection of the real world seems to be substantially simplified.*

**Response 1:** Thank you for pointing this out. To justify the assumptions, I have cited references 29 and 30 in lines 202 to 205 of the manuscript to explain their validity.

***Q2:*** *Compare with existing literature, both in terms of approach/method and results. Also check bold statements made about gaps in current literature.*

**Response 2:** Apologies for this issue. I have revised the literature review section to refine statements about existing studies and have cited references 26, 27, and 28 to ensure a more comprehensive comparison. Additionally, I have restated the contributions of this study and its advantages over existing research in lines 180 to 197 of the manuscript.

***Q3:*** *Highlight limitations of the presented work.*

**Response 3:** Thank you for your suggestion. I have added a discussion on the limitations of this study in the "Conclusion" section, specifically in lines 629 to 637, to ensure the objectivity and completeness of the research findings.

Respond to Reviewer #1’ comments

***Q1:*** *If it is not required by the journal, it is recommended to mark the number of parts of the article with the serial number before each chapter. Otherwise, it will appear that the structure of the article is somewhat chaotic.*

**Response 1:** Thank you for your insightful suggestion. I have added numbering to the sections of the paper to improve its structure.

***Q2:*** *How are some indicators, such as Queuing pattern, specifically defined? The article seems to have omitted this information.*

**Response 2:** Thank you for pointing out this problem. I have now clarified the definition of the indicators in Section 3.1 of the article. Specifically, from lines 232 to 239, the queuing pattern is explained as the specific queuing order of the two types of vehicles in a mixed traffic flow containing CAVs (Connected Autonomous Vehicles) and HVs (Human-driven Vehicles). The queuing patterns can be categorized into three types: the first type, where all CAVs are positioned at the front of the queue; the second type, where CAVs and HVs are randomly mixed within the queue; and the third type, where all HVs are positioned at the front of the queue.

***Q3:*** *The manuscript assumes all vehicles run along traffic lanes. However, the vehicular movement inside intersections is two-dimensional. This assumption should be clarified. The relevant existing studies should be cited. E.g., Microscopic traffic modeling inside intersections Interactions between drivers, Transportation Science, 2023; Unprotected left-turn behavior model capturing path variations at intersections, IEEE-T-ITS, 2023.*

**Response 3:** Thank you for your valuable suggestion. I have addressed this point in the manuscript by citing two relevant studies in lines 202 to 205 to explain the rationale behind assuming that all vehicles run along traffic lanes.

***Q4:*** *The authors like to use large paragraphs to describe a certain problem, but the logical structure of the language expression is not clear enough. For example, in the section on influencing factors, the authors use a long paragraph to explain the various factors that need to be considered when formulating traffic strategies, but the overall structure is somewhat loose and lacks a clear logical thread. It is recommended that the authors split this paragraph into several sections, each focusing on one or several related factors to make the content clearer and easier to understand.*

**Response 4:** Thank you for your thoughtful feedback. I have revised the manuscript by splitting the paragraph into several sections in Section 3.1, from lines 225 to 242, each focusing on one or more related factors. Your suggestion has greatly improved the clarity and readability of the manuscript.

***Q5:*** *Only one comparison is used, which doesn't seem convincing enough. More experiments are suggested.*

**Response 5:** We are grateful for your constructive suggestions. I have added a comparison experiment using the First-Come, First-Served (FCFS) strategy in Section 4.2.2 and updated Figures 5 and 6 to make the research results more convincing. The results indicate that the optimized order of passage significantly reduces per capita delay and travel time compared to both actuated control and FCFS.

Respond to Reviewer #2’ comments

***Q1:*** *The literature review is incomplete about mixed traffic control. Recent works have been dedicated to the optimization and analysis of traffic intersections under mixed traffic scenarios and have not been referenced. You state that “…researches involving the passage order of intersections in mixed traffic environments consider a single optimization objective and are unable to weigh the …”. This is not quite true. See for example,*

*Changxin Wan, Xiaonian Shan, Peng Hao, Guoyuan Wu, Multi-objective coordinated control strategy for mixed traffic with partially connected and automated vehicles in urban corridors, Physica A: Statistical Mechanics and its Applications, Volume 635, 2024.*

*You should clearly identify the limitations of previous and recent works about the same subject and how do you expect to overcome some of their potential limitations.*

**Response 1:** Thank you very much for pointing out this mistake. I have added relevant literature on intersection passage strategies in mixed traffic environments in Section 2, from lines 166 to 177 (references 26, 27, and 28). Additionally, in lines 180 to 197, I have explained how we overcome the limitations of these studies.

***Q2:*** *How do you control the order of passage? The intersections do not have traffic signals. So, how do you control the movement of human-driven vehicles?*

**Response 2:** Apologies for the oversight in the manuscript. The intersections in this study are signalized intersections, and we control the order of passage by switching the signal phases to determine the sequence in which vehicles from different approaches pass through the intersection. I have added this clarification in lines 210 to 211 of the manuscript.

***Q3:*** *Why is the travel time reducing as the penetration rate increase?*

**Response 3:** Thank you for raising this question. CAVs (Connected Autonomous Vehicles) have shorter reaction times and can communicate with each other, which enhances coordination between vehicles and reduces travel time. AVs are able to maintain optimal speeds and spacing, minimizing delays caused by unstable driving behaviors. Therefore, as the penetration rate of autonomous vehicles increases, travel time will decrease. I have added an explanation regarding this in lines 334 to 338 of the manuscript.

***Q4:*** *In tables 11 and 12, what is the minimum distance between human-driven and autonomous vehicles?*
**Response 4:** Thank you for raising this question, and I apologize for not providing a clear explanation of the definition of ‘*minGap*’. ‘*minGap*’ refers to the minimum distance between a vehicle and the vehicle in front when the vehicle is at a stop. This definition has been updated in Tables 11 and 12. Therefore, when a CAV follows an HV, the minimum distance between the two vehicles is 0.5 meters, and when an HV follows a CAV, the minimum distance is 1 meter.

***Q5:*** *The model defines the passing order of vehicles based on intersection scenarios. Are you considering the same conditions for all directions? It is not clear how you determine the order of passage from the traffic scenario. Is the order fixed during the complete simulation? When do the system decide to switch to a different traffic phase?*

**Response 5:** Thank you for raising these valuable questions. Based on the data from the five experiments in Table 13, there are differences in Average speed, Queuing pattern, Automated vehicle penetration, Number of cars, Number of buses, and Left-turn rate for all directions. The passing order is determined by comparing the comprehensive efficiency of each direction, calculated using Data Envelopment Analysis (DEA), with the direction having higher efficiency prioritized for passage. Since each simulation cycle corresponds to one platoon passing through each approach and only one cycle is performed in each simulation, the order of passage remains fixed throughout the simulation. I have added this clarification in lines 596 to 597 of the manuscript. Regarding the switching to different traffic phases, vehicles with a headway of less than 3 seconds are grouped into a single platoon, and each signal cycle allows one platoon to pass through each approach. The passing order is recalculated for each cycle. I have added this explanation in lines 211 to 213 of the manuscript.

***Q6:*** *What do you consider to be the traditional actuated control mechanisms?*

**Response 6:** Thank you for raising this important question. In SUMO, actuated signal control dynamically adjusts traffic signal phases by continuously monitoring vehicle flow in real-time. This mechanism relies on vehicle detectors installed at the intersection, which collect flow data from each lane and transmit it to the signal control system. Based on this data, the system determines whether to extend or shorten the green light duration for specific directions, thereby optimizing intersection efficiency. To make the content clearer, I have added this explanation in lines 588 to 592 of the manuscript.

***Q7:*** *How do you compare with other optimization strategies?*

**Response 7:** I appreciate your interest in this matter. In comparison with other optimization strategies, the contribution of this study lies in the proposed optimization model, which comprehensively considers various factors, including average speed, number of cars, penetration of automated vehicles, queuing pattern, left-turn rate, and number of buses. This model provides a more holistic representation of the complex traffic flow characteristics at intersections. Additionally, the optimization objectives focus on improving intersection passage efficiency, incorporating multi-dimensional goals such as per capita delay, travel time, and traffic volume, thereby making the optimization results more comprehensive and accurate. These influencing factors and optimization objectives provide a more precise and scientifically grounded basis for optimizing intersection passage efficiency. Regarding the advantages over other optimization strategies, I have added supplementary information in lines 187 to 197 of the manuscript.

***Q8:*** *How do you compare against intelligent solutions based, for example, on reinforcement learning?*

**Response 8:** Thank you for your insightful question. Undoubtedly, intelligent solutions, especially those based on reinforcement learning, have become quite popular in the field of intelligent transportation. However, methods based on reinforcement learning rely on large-scale data training, and the complexity of traffic environments may make it difficult to accurately map the states, actions, and rewards in reinforcement learning, potentially resulting in poor training outcomes. In contrast, the method proposed in this study effectively handles complex traffic environments without the need for large-scale data training, quickly and accurately determining the optimal passage order for each direction at the intersection. To highlight the contribution of this study, I have added this content in lines 184 to 187 of the manuscript.

We sincerely appreciate the reviewers’ insightful feedback, which has significantly improved our manuscript. We look forward to your response and appreciate your time and consideration.

Your faithfully

On behalf of co-authors (Professor Dr Jiajun Shen; Guanyu Fu; Yu Wang)

Zhipeng Zhou

College of Architectural Science and Engineering, Yangzhou University,

E-mail: zzpash@outlook.com
