# Supplementary material for: Intersection passing strategies for human-driven and autonomous vehicles in mixed traffic using DEA
Source: PLoS One. 2025 Apr 29;20(4):e0321566. doi: 10.1371/journal.pone.0321566 (PMC12040198; doi:10.1371/journal.pone.0321566)
Supplement: Attachment — Submitted filename: Response_to_Reviewers_auresp_2.docx [file pone.0321566.s003.docx]

**Response to Reviewers**

**Manuscript number:** PONE-D-24-52478

**Title:** Intersection passing strategies for human-driven and autonomous vehicles in mixed traffic using DEA

Dear editor and reviewers,

Thank you for giving us the opportunity to revise and resubmit our manuscript entitled “Intersection passing strategies for human-driven and autonomous vehicles in mixed traffic using DEA” (ID: PONE-D-24-52478). We sincerely appreciate the reviewers' constructive feedback and have carefully addressed all comments. According to your nice suggestions, we have made extensive corrections to our previous manuscript, the detailed corrections are listed below.

We sincerely thank the editor and all reviewers for their valuable feedback that we have used to improve the quality of our manuscript. The reviewer comments are laid out below in italicized font and specific concerns have been numbered. Our response is given in normal font and changes/ additions to the manuscript are given in the yellow text.

Respond to additional editor comments:

***Q1:*** *Code of the proposed solution should be made available. At least to the reviewer, and preferably to the public, to ensure reproducibility.*

**Response 1:** We appreciate the comment regarding the availability of code for reproducibility. In our study, all results are derived directly from the formulas in Section 3.3 (Formulas 3-16 to 3-24) of the paper. These formulas do not require computational algorithms to obtain the results, and thus, no code is necessary for the study. Therefore, we are unable to provide code for this research.

***Q2:*** *Relationship between the traffic lights and the metrics.*

**Response 2:** We acknowledge the importance of the dynamics of traffic lights in traffic flow analysis. However, our study primarily focuses on determining the optimal intersection passage order under varying traffic demands, and as such, traffic signal timing was not considered within the scope of this work. We recognize the significant impact of traffic signal timing on overall intersection efficiency, and this could be a direction for future research. We have added a discussion of this limitation on lines 638 to 641 in the manuscript, and we hope to explore this aspect in future studies.

***Q3:*** *Comparison with existing approaches required.*

**Response 3:** We thank Reviewer #2 for suggesting a comparison with deep reinforcement learning (DRL) methods. In existing research, many DRL-based approaches have been applied to traffic signal control and intersection optimization, where reinforcement learning agents automatically adjust traffic signals to optimize traffic flow. However, our study proposes an optimization method that directly calculates the intersection passage order based on traffic flow conditions, focusing on determining the optimal passage order. Future research could combine our approach with DRL methods to optimize traffic signals at intersections.

Respond to Reviewer #2’ comments

***Q1:*** *To ensure reproducibility of the results, the code of the proposed solution should be made public on a website.*

**Response 1:** Thank you for raising this important point regarding reproducibility. In our study, all results are derived directly from formulas 3-16 to 3-24 in Section 3.3 of the paper. These formulas do not require computational algorithms to obtain the results. Therefore, no code can be provided for this study.

***Q2:*** *The dynamics of traffic lights should be included, that is, the green, red and yellow times and how they affect the metrics.*

**Response 2:** Thank you for the detailed feedback on our research. Regarding the traffic signal timing issue, our study currently focuses on determining the optimal intersection passage order under different traffic demands, and thus, traffic signal timing is not considered within the scope of this study. We recognize that traffic signal timing plays an important role in the overall traffic efficiency of intersections, and it can be considered as a direction for future research. I have added a discussion of this limitation in lines 638 to 641 of the manuscript, and we hope to explore it in future studies.

***Q3:*** *Complex or not, approaches based on DRL exist with good results. The work should be compared with these approaches.*

**Response 3:** Thank you for your valuable feedback. It is undeniable that many deep reinforcement learning (DRL)-based methods have been applied to traffic signal control and intersection throughput optimization in existing research. These methods typically rely on large-scale data training, where reinforcement learning agents automatically adjust traffic signal timing to optimize traffic flow and reduce vehicle delays. The focus of these studies is often on long-term decision-making, such as dynamically adjusting the signal cycle and duration, and they tend to prioritize optimizing signal control rather than directly determining the passage order.

However, this study proposes an optimization method that directly calculates the intersection passage order based on traffic flow conditions. This method focuses on determining the optimal passage order using real-time traffic flow data and specific traffic demand.

Although the focus of this study is on directly calculating the intersection passage order based on traffic flow conditions, without involving specific signal timing research, future studies may consider applying DRL methods to optimize signal timing. Combining this with the current passage order optimization could further enhance the overall throughput of intersections.

***Q4:*** *The units are missing in the graphs. For example, in Figures 5 and 6, what are the time units?*
**Response 4:** Thank you for pointing out the issue with the missing units in the graphs. Regarding Figures 5 and 6, I have made the necessary revisions and added the appropriate time units to the graphs. Thank you again for your careful review.

We sincerely appreciate the reviewers’ insightful feedback, which has significantly improved our manuscript. We look forward to your response and appreciate your time and consideration.

Your faithfully

On behalf of co-authors (Professor Dr Jiajun Shen; Guanyu Fu; Yu Wang)

Zhipeng Zhou

College of Architectural Science and Engineering, Yangzhou University,

E-mail: zzpash@outlook.com
